# Comparison of immunotherapy based total neoadjuvant therapy or standard neoadjuvant chemoradiation for locally advanced rectal cancer: a multi-institutional retrospective study

**DOI:** 10.3389/fimmu.2025.1513716

**Published:** 2025-04-14

**Authors:** Wen Zhao, Wenxing Gao, Jitao Du, Dingchang Li, Xianqiang Liu, Zhengyao Chang, Peng Chen, Xu Sun, Yingjie Zhao, Hanqing Jiao, Xiangbin Wan, Guanglong Dong

**Affiliations:** ^1^ School of Medicine, Nankai University, Tianjin, China; ^2^ Department of General Surgery, the First Medical Center of Chinese PLA General Hospital, Beijing, China; ^3^ Medical School of Chinese PLA, Beijing, China; ^4^ Department of General Surgery, the Affiliated Cancer Hospital of Zhengzhou University, Zhengzhou, China; ^5^ Department of General Surgery, the Fifth Medical Center of Chinese PLA General Hospital, Beijing, China; ^6^ Department of General Surgery, the Eighth Medical Center of Chinese PLA General Hospital, Beijing, China

**Keywords:** total neoadjuvant treatment, locally advanced rectal cancer, pathological complete response, downstaging, survival

## Abstract

**Background:**

Combining radiation therapy with immunotherapy produces a synergistic effect in patients with microsatellite stable/mismatch repair-proficient (MSS/pMMR) locally advanced rectal cancer (LARC). This study aimed to evaluate the long-term outcomes and safety of immunotherapy combined with long-course chemoradiotherapy (ICIs + nCRT) versus immunotherapy combined with total neoadjuvant therapy (ICIs + TNT).

**Methods:**

This retrospective study collected clinical data of adult patients with clinical T3-4 and/or N1 rectal adenocarcinoma who underwent ICIs + TNT or ICIs + nCRT followed by curative surgery at four medical centers between March 2020 and August 2021. The study compared clinical efficacy, disease-free survival (DFS), overall survival (OS) at 3 years postoperatively, and adverse event.

**Results:**

Among 211 enrolled patients, 89 (42%) received ICIs + TNT, while 122 (58%) underwent ICIs + nCRT, with a median age of 56.0 years (range, 20.0-75.0 years). The ICIs + TNT group had a higher median number of resected lymph nodes (15.0 [range, 4.0-37.0] vs. 13.0 [range, 3.0-33.0], *P*=0.028) compared to the ICIs+nCRT group. However, the groups had no substantial difference in median operative time. The pathological complete response (pCR) rate was 49.4% (44/89, 95% confidence interval [CI] 39.8%-61.3%) in the ICIs + TNT group compared to 35.3% (43/122, 95% CI 26.8%-44.4%) in the ICIs + nCRT group, respectively, with significant difference (*P*=0.039). After adjusting for potential confounders, the 3-year DFS rates were comparable between the two groups (84.3% vs. 81.9%; *P*=0.620), as were the OS rates (94.0% vs. 91.1%; *P*=0.634). Factors independently associated with poorer DFS included age ≤50 years (*P*=0.044) and a neoadjuvant rectal (NAR) score ≥8 (*P*=0.008). Similarly, patients aged ≤50 years (*P*=0.025) exhibited a trend toward worse OS than those older than 50 years. The safety profiles of the two treatment groups were similar.

**Conclusions:**

Overall, ICIs + TNT demonstrated therapeutic efficacy and a safety profile comparable to ICIs + nCRT in patients with LARC and MSS/pMMR status. Although ICIs + TNT achieved numerically higher downstaging rates, it was not associated with improved survival outcomes. These findings underscore the importance of refining patient selection criteria and making judicious treatment decisions to enhance the prognosis of individuals with rectal cancer.

## Introduction

For patients diagnosed with locally advanced rectal cancer (LARC), the standard treatment strategy involves neoadjuvant chemoradiotherapy (nCRT) followed by total mesorectal excision (TME) and adjuvant chemotherapy (1, 2). Preoperative chemoradiotherapy has proven effective in targeting primary tumors and micrometastases, enhancing tumor regression, and diminishing the risk of local recurrence (3–5). However, this approach has limitations, including a low pathological complete response (pCR) rate of 15%-20% and minimal improvement in sphincter preservation rates (6). In addition, the prolonged interval between diagnosis and surgery, along with the delayed administration of systemic chemotherapy, which elevates the risk of distant metastasis (7). In the context of numerous studies aimed at enhancing the effectiveness of neoadjuvant therapy and improving compliance with perioperative systemic chemotherapy, total neoadjuvant therapy (TNT) has emerged as a significant approach. Currently, TNT is regarded as the standard treatment for LARC and is endorsed by the National Comprehensive Cancer Network (NCCN) clinical practice guidelines (8).

In recent years, immunotherapy, particularly immune checkpoint inhibitors (ICIs), has revolutionized the treatment landscape for various tumors (9), offering substantial efficacy and reshaping traditional therapeutic paradigms. For patients with microsatellite instability-high/deficient mismatch repair (MSI-H/dMMR) LARC, neoadjuvant immunotherapy has significantly improved clinical complete response (cCR) rates. Conversely, individuals with microsatellite stable/mismatch repair-proficient (MSS/pMMR) tumors derive limited benefit from ICI monotherapy. This finding underscores the urgent need to explore more effective neoadjuvant or TNT regimens for these patients. Neoadjuvant immunotherapy for LARC has garnered increasing attention in recent studies, with current approaches primarily focusing on two modalities: neoadjuvant immunotherapy combined with chemoradiotherapy (ICIs + nCRT) and neoadjuvant immunotherapy combined with TNT (ICIs + TNT). Recently, several clinical trials have reported the efficacy and safety of ICIs + TNT and ICIs + nCRT regimens in patients with MSS/pMMR LARC (10–12). Both neoadjuvant immunotherapy approaches demonstrated favorable tumor regression and clinical potential, as pCR of 50.0% observed with long-course chemoradiotherapy (LCRT) in conjunction with concurrent tislelizumab (10), and pCR of 33.3% observed with TNT in combination with induction ICIs and chemotherapy, followed by LCRT (12). However, most studies exploring the combination of chemoradiotherapy and immunotherapy are phase II, single-arm trials with limited sample sizes, leaving the long-term oncologic outcomes uncertain.

No studies directly compare the safety and efficacy of different preoperative immunotherapy modalities to determine the optimal neoadjuvant immunotherapy regimen for LARC. Against this background, a multi-institutional retrospective analysis was performed to evaluate and compare the efficacy and safety of ICIs combined with TNT (ICIs + TNT) versus ICIs combined with nCRT (ICIs + nCRT) for treating LARC.

## Materials and methods

### Patient selection

This study retrospectively analyzed databases from four centers within the Chinese PLA General Hospital and the Affiliated Cancer Hospital of Zhengzhou University. Patients with LARC who underwent neoadjuvant immunotherapy in combination with TNT or nCRT, followed by TME, between March 2020 and August 2021, were included in the study. The inclusion criteria were as follows: 1) histopathologically confirmed rectal adenocarcinoma with a baseline clinical stage of T3-T4 or any T stage with lymph node involvement; 2) tumor located ≤10 cm from the anal verge; 3) absence of distant metastasis; 4) patient age 18-75 years; 5) no anti-tumor therapy before enrollment. Patients with unresectable advanced or metastatic tumors or incomplete clinical data were excluded from the analysis. This research was approved by the ethics committees of participating institutions, and written informed consent was obtained from all participants before inclusion.

Expression of the key four MMR proteins, namely, mutL Homolog 1 (MLH1), mutS Homolog 2 (MSH2), mutS Homolog 6 (MSH6), and postmeiotic segregation increased 2 (PMS2), was evaluated using MMR immunohistochemistry (IHC). Moreover, microsatellite instability (MSI) testing was performed on genomic DNA using polymerase chain reaction (PCR)-based methods. Only patients with MSS or pMMR status were included in this study.

### Neoadjuvant treatment schedules

Patients in the ICIs+TNT group were treated using a consolidation approach for TNT. This regimen included LCRT (50.4 Gy in 28 fractions combined with concurrent oral capecitabine 825 mg/m^2^ twice daily) alongside programmed cell death protein 1 (PD-1) inhibitors. Subsequently, patients received two cycles of capecitabine and oxaliplatin (CAPOX) in combination with PD-1 inhibitors. Patients in the ICIs + nCRT group received LCRT (50.4 Gy in 28 fractions combined with CAPOX) and three cycles of PD-1 inhibitors concurrently as the neoadjuvant therapy. The clinical target volume (CTV), planning target volumes (PTV) and organs at risk (OARs) were delineated by the senior radiation oncologist. The technique employed in four centers to deliver preoperative radiotherapy treatments was three-dimensional conformal radiotherapy (3D-CRT). The Accuray Radixact tomotherapy system (Accuray Inc., Madison, WI, USA) were used for treatment planning. Postoperative adjuvant treatment commenced 4 to 6 weeks postoperatively. The PD-1 inhibitors used across both groups included camrelizumab, sintilimab, pembrolizumab, tislelizumab, nivolumab, and durvalumab.

Radical surgery was recommended for patients deemed eligible for R0 resection following neoadjuvant therapy. Surgical procedures were conducted by TME principles.

### Follow-up

Dedicated personnel at each medical center managed patient follow-up, conducted every three months during the first two years following radical surgery and every 6 months thereafter until death or study termination. Follow-up assessments included physical examinations, colonoscopy, and imaging studies such as computed tomography (CT) scans of the chest, abdomen, pelvis, and rectal magnetic resonance imaging (MRI). The following parameters were documented during the follow-up period: time to disease progression or death; radiological tumor evaluation; incidence and severity of adverse events (AEs) during the study; and patient survival status (with telephone follow-ups conducted as required).

### Assessments

The primary endpoint of this study was the pCR rate, defined as the absence oftumor cells in the primary tumor and lymph nodes following radical surgery (ypT0N0M0). Secondary endpoints included the incidence of grade 3-4 acute toxicity, objective response rate (ORR), 3-year disease-free survival (DFS) and overall survival (OS). Radiographic response was assessed every two cycles of PD-1 inhibitor therapy using the revised RECIST guidelines (version 1.1) (13). Postoperative pathological tumor regression was evaluated according to the 8th edition of the American Joint Committee on Cancer (AJCC) guidelines. tumor regression grade (TRG) 0: no viable tumor cells remaining; TRG 1: individual or small clusters of tumor cells; TRG 2: tumor remnants with substantial quantities of fibrotic mesenchyme visible; TRG 3: extensive residual cancer with minimal evidence of tumor regression. DFS was defined as the time from definitive surgery to the first tumor recurrence or death. OS was defined as the time from definitive surgery until death from any cause.

### Statistical analysis

All statistical analyses of the data were conducted with R software (Version: 4.3.0). Categorical variables were presented using frequencies and percentages, while continuous variables were presented using medians and ranges. When the continuous data did not follow a normal distribution, the nonparametric test was employed. The differences in the distribution of categorical variables between the two groups were compared by employing the χ^2^ test or Fisher’s exact test. Survival analysis was conducted using the Kaplan-Meier method, and the significance of DFS and OS were determined by Log-rank test. Univariate and multivariate logistic regression models were used to investigate the factors of sociodemographic and clinical variables in relation to pCR. Cox proportional hazards models were employed to estimate the effect of ICIs+TNT or ICIs+nCRT on prognosis while adjusting for the potential confounding factors. The estimated effects of covariates were employed to calculate odds ratios (ORs) or hazard ratios (HRs), accompanied by 95% confidence intervals (CIs). All statistical analyses were two-tailed, and *P* < 0.05 was considered statistically significant.

## Results

### Patient characteristics

**Figure 1:**
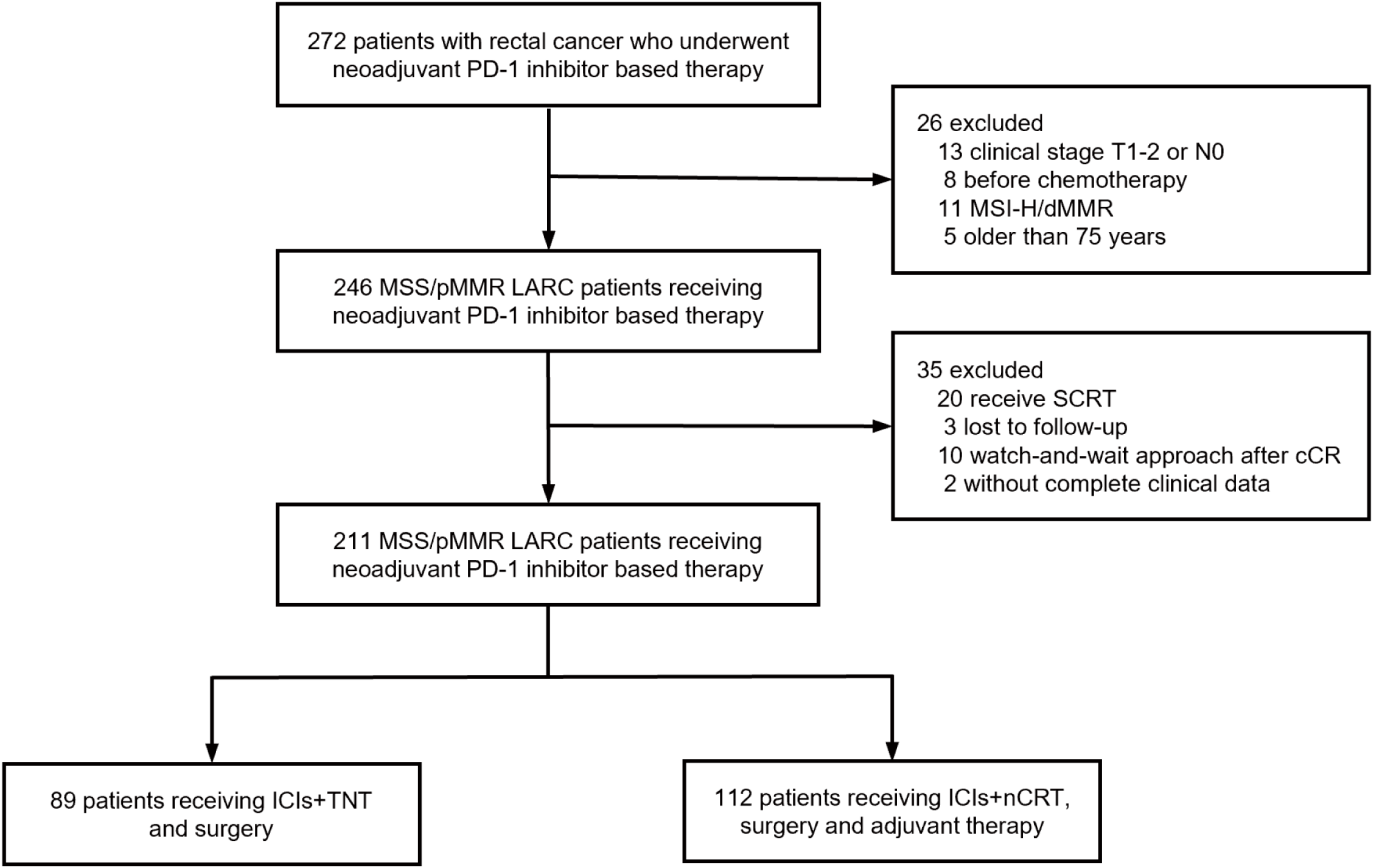
The flowchart of enrolled patients in this study.

Between March 2020 and August 2021, 247 patients with LARC were recruited from four medical centers in Beijing and Zhengzhou, China. Patients achieving cCRunder non-operative management or lacking follow-up date were excluded. A total of 211 patients with MSS/pMMR LARC were included in the final analysis, comprising 89 patients in the ICIs + TNT group and 122 in the ICIs + nCRT group (**Figure 1**). Of the cohort, 32.2% were female, 74.9% presented with clinical stage III disease, and 78.2% had tumors located ≤5 cm from the anal verge. Baseline patient and tumor characteristics were comparable between the two groups (**Table 1**).

**Table 1:** Patients baseline characteristics.

Variables	Overall (n=211)	ICIs+TNT (n=89)	ICIs+ nCRT (n=122)	*P* value
Age (years)				0.256
Mean (SD)	57.2 (10.8)	58.2 (10.2)	56.5 (11.2)	
Sex				0.694
Male	143 (67.8%)	59 (66.3%)	84 (68.9%)	
Female	68 (32.2%)	30 (33.7%)	38 (31.1%)	
BMI				0.544
Mean (SD)	24.2 (3.2)	24.3 (3.6)	24.0 (3.3)	
Smoking status				0.227
Nonsmoker	156 (73.9%)	62 (69.7%)	94 (77.0%)	
Smoker	55 (26.1%)	27 (30.3%)	28 (23.0%)	
ECOG PS				0.253
0	155 (73.5%)	69 (77.5%)	86 (70.5%)	
1	56 (26.5%)	20 (22.5%)	36 (29.5%)	
Histologic grade				0.167
Poorly differentiated	56 (26.5%)	28 (31.5%)	28 (23.0%)	
Moderately or well differentiated	155 (73.5%)	61 (68.5%)	94 (77.0%)	
Tumor location				0.417
Low (≤5 cm)	165 (78.2%)	72 (80.9%)	93 (76.2%)	
Mid (>5-10 cm)	46 (21.8%)	17 (19.1%)	29 (23.8%)	
Clinical T stage				0.380
T2	12 (5.7%)	3 (3.4%)	9 (7.4%)	
T3	93 (44.1%)	38 (42.7%)	55 (45.1%)	
T4	106 (50.2%)	48 (53.9%)	58 (47.5%)	
Clinical N stage				0.485
N0	55 (26.1%)	21 (23.6%)	34 (27.9%)	
N1-2	156 (73.9%)	68 (76.4%)	88 (72.1%)	
CEA level				0.802
<5 ng/ml	163 (77.3%)	68 (76.4%)	95 (77.9%)	
≥5 ng/ml	48 (22.7%)	21 (23.6%)	27 (22.1%)	
*RAS* status				0.601
Mutant-type	60 (28.4%)	27 (30.3%)	33 (27.0%)	
Wild-type	151 (71.6%)	62 (69.7%)	89 (73.0%)	
MRF				0.517
Negative	138 (65.4%)	56 (62.9%)	82 (67.2%)	
Positive	73 (34.6%)	33 (37.1%)	40 (32.8%)	
EMV1				0.804
Negative	157 (74.4%)	67 (75.3%)	90 (73.8%)	
Positive	54 (25.6%)	22 (24.7%)	32 (26.2%)	
PD-L1 expression				0.764
CPS <1	57 (27.0%)	25 (28.1%)	32 (26.2%)	
CPS ≥1	154 (73.0%)	64 (71.9%)	90 (73.8%)	

BMI, body mass index; ECOG PS, Eastern Cooperative Oncology Group performance status, MRF, mesorectal fascia; EMVI, extramural venous invasion; CEA, carcinoembryonic antigen; CPS, combined positive score.

### Efficacy of two neoadjuvant treatments

Among the 246 MSS/pMMR LARC patients who received neoadjuvant immunotherapy initially enrolled in this study, cCR was confirmed in 3 and 7 patients in the ICIs + TNT and ICIs + nCRT group, respectively. Of the 10 patients with cCR were managed with a watch-and-wait (W&W) approach after treatment completion. The median time to reach cCR was 4.8 months (range, 1.6-10.2 months) and 5.5 months (range, 4.0-11.6 months) in the ICIs + TNT and ICIs + nCRT group, respectively. All these 10 cCR patients were still managed with a W&W approach without tumor regrowth or metastasis with at least 28.2 months’ follow-up.

Pathological staging was performed for all 211 patients, and efficacy outcomes are summarized in **Table 2**. Across the cohort, 81.0% achieved an ORR based on radiological assessment, with 32.7% showing complete response (CR) and 48.3% partial response (PR). The median time to response (TTR) in the ICIs + TNT group was 1.9 months (interquartile range [IQR] 1.3-2.7 months), significantly shorter than 2.5 months (IQR 1.5-3.2 months) in the ICIs + nCRT group. Tumor regression grades (TRG 0-1) were significantly higher in the ICIs + TNT group compared to the ICIs + nCRT group (80.9% vs 68.0%, *P*=0.037). However, no significant difference in ORR was observed between the two regimens (*P*=0.083).

**Table 2:** Tumor response of two neoadjuvant treatments.

Variables	Overall (n=211)	ICIs+TNT (n=89)	ICIs+ nCRT (n=122)	*P* value
TTR, median (IQR), mo	2.2 (1.4-2.8)	1.9 (1.3-2.7)	2.5 (1.5-3.2)	0.016
ORR	171 (81.0%)	77 (86.5%)	94 (77.0%)	0.083
DCR	207 (98.1%)	88 (98.9%)	119 (97.5%)	0.640
Radiographic response				0.205
CR	69 (32.7%)	35 (39.3%)	34 (27.9%)	
PR	102 (48.3%)	42 (47.2%)	60 (49.2%)	
SD	36 (17.1%)	11 (12.4%)	25 (20.5%)	
PD	4 (1.9%)	1 (1.1%)	3 (2.5%)	
TRG				0.096
TRG 0 (ypT0N0M0)	87 (41.2%)	44 (49.4%)	43 (35.3%)	
TRG 1	67 (31.8%)	28 (31.5%)	39 (32.0%)	
TRG 2	48 (22.7%)	15 (16.9%)	33 (27.0%)	
TRG 3	9 (4.3%)	2 (2.2%)	7 (5.7%)	
Combined TRG 0-1	149 (70.6%)	72 (80.9%)	83 (68.0%)	0.037

IQR, interquartile range; ORR, objective response rate; DCR, disease control rate; CR, complete response; PR, partial response; SD, stable disease; PD, progression disease.

### Surgical and pathological outcomes

**Figure 2:**
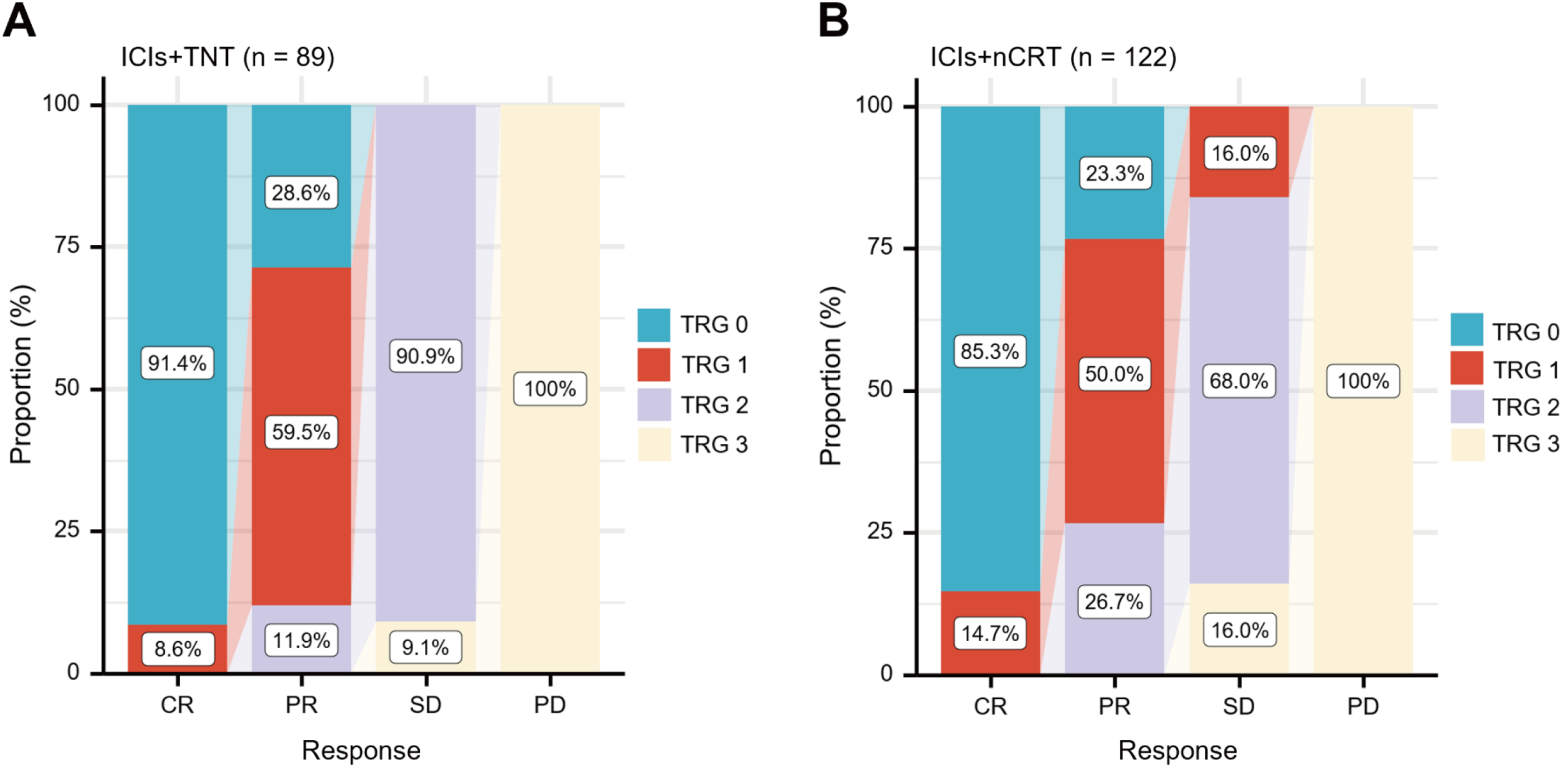
Relationship between radiologic response and tumor regression grade (TRG). **(A)** TRG among patients with different radiologic responses in the ICIs + TNT group (n = 89). **(B)** TRG among patients with different radiologic responses in the ICIs + nCRT group (n = 122). CR, complete response; PR, partial response; SD, stable disease; PD, progressive disease.

**Figure 3:**
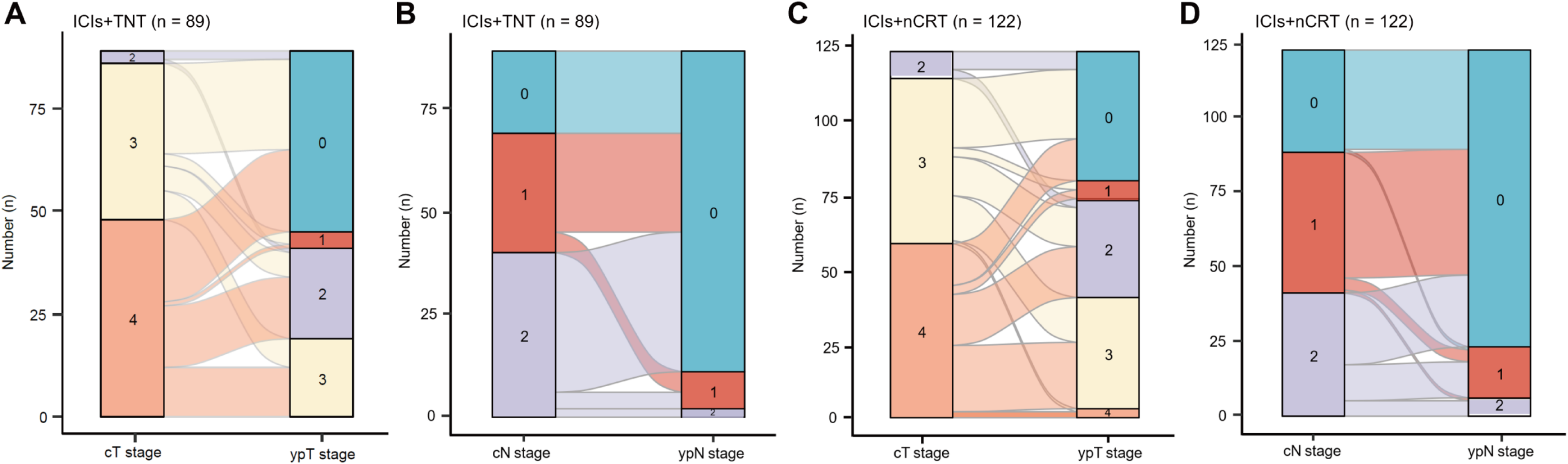
Clinical efficacy after neoadjuvant. **(A, B)** Changes of pre-treatment cT and cN stage to post-treatment ypT and ypN stage in 89 patients who reached the primary endpoint in the ICIs + TNT group. **(C, D)** Changes of pre-treatment cT and cN stage to post-treatment ypT and ypN stage in 122 patients who reached the primary endpoint in the ICIs + nCRT group.

All 211 patients underwent TME. Details regarding the type of surgery, median operation duration, surgical resection status, and postoperative pathological tumor stage are presented in **Table 3**. The median interval between neoadjuvant therapy and the operation was 2.32 months (IQR 2.04-2.48) in the ICIs + TNT group and 2.21 months (IQR 1.96-2.36) in the ICIs + nCRT group. The R0 resection rates were 98.9% (88/89) in the ICIs + TNT group and 97.5% (119/122) in the ICIs + nCRT group. Sphincter-preserving surgery was performed in 79 (88.8%) and 102 (83.6%) patients, respectively. A significantly higher proportion of patients in the ICIs + TNT group achieved lower pathological tumor stages (ypT0-2) compared to the ICIs + nCRT group (79.8% vs. 67.2%, *P*=0.044). Similarly, the pCR (ypT0N0M0) rate was significantly greater in the ICIs + TNT group (49.4%) compared to the ICIs+nCRT group (35.3%) (*P*=0.039). Among patients with PR, pCR was observed in 27.3% of those in the ICIs + TNT group and 32.6% of those in the ICIs + nCRT group, although this difference was not statistically significant. However, no pCR cases were identified among patients with stable disease (SD) or progressive disease (PD) in either group (**Figure 2**). Regarding tumor downstaging, 80 patients (89.9%) in the ICIs + TNT group and 102 patients (83.6%) in the ICIs + nCRT group exhibited clinical T-stage downstaging, with no substantial difference (*P*=0.191). Similarly, downstaging of the clinical N stage was observed in 62 patients (69.7%) in the ICIs + TNT group and 78 (63.9%) in the ICIs + nCRT group (**Figure 3**).

**Table 3:** Surgical and pathological characteristics.

Variables	Overall (n=211)	ICIs+TNT (n=89)	ICIs+ nCRT (n=122)	*P* value
Type of surgery				0.602
Anterior resection	181 (85.8%)	79 (88.8%)	102 (83.6%)	
Abdominoperineal resection	27 (12.8%)	9 (10.1%)	18 (14.8%)	
Hartmann’s procedure	3 (1.4%)	1 (1.1%)	2 (1.6%)	
Resection grade				0.640
R0	207 (98.1%)	88 (98.9%)	119 (97.5%)	
R1-2	4 (1.9%)	1 (1.1%)	3 (2.5%)	
Pathological tumor stage (ypT)				0.159
ypT0	87 (41.2%)	44 (49.4%)	43 (35.2%)	
ypT1	10 (4.7%)	4 (4.5%)	6 (4.9%)	
ypT2	56 (26.5%)	23 (25.8%)	33 (27.0%)	
ypT3	55 (26.1%)	18 (20.2%)	37 (30.3%)	
ypT4	3 (1.4%)	0 (0.0%)	3 (2.5%)	
Combined ypT				0.044
Combined ypT0-2	153 (72.5%)	71 (79.8%)	82 (67.2%)	
Combined ypT3-4	60 (27.5%)	18 (20.2%)	40 (32.8%)	
Pathological node stage (ypN)				0.466
ypN0	177 (83.9%)	78 (87.6%)	99 (81.1%)	
ypN1	26 (12.3%)	9 (10.1%)	17 (13.9%)	
ypN2	8 (3.8%)	2 (2.2%)	6 (4.9%)	
Median operation time (range), h	3.4 (1.6-9.0)	3.5 (1.7-9.0)	3.2 (1.6-8.6)	0.113
Sphincter-sparing surgery				0.289
Yes	181 (85.8%)	79 (88.8%)	102 (83.6%)	
No	30 (14.2%)	10 (11.2%)	20 (16.4%)	
Median number of lymph nodes resected (range)	13 (3-37)	15 (4-37)	13 (3-33)	0.028

Univariate analysis identified several factors positively correlated with pCR, including age >50 years, earlier clinical T stage, CEA levels <5 ng/ml, PD-L1 combined positive score (CPS) ≥2, and treatment with ICIs + TNT. However, PD-L1 CPS ≥1 was not significantly correlated with increased odds of pCR. Multivariate logistic regression analysis further revealed that age and the neoadjuvant immunotherapy regimen (ICIs + TNT vs. ICIs + nCRT) were independent predictors of pCR after adjusting for confounding variables (**Supplementary Table 1**). Patients aged ≤50 years were 32% less likely to achieve pCR compared to those aged >50 years (OR: 0.320, 95% CI: 0.142-0.725, *P*=0.006). Moreover, ICIs + TNT demonstrated a significantly higher likelihood of pCR than ICIs + nCRT (OR: 2.170, 95% CI: 1.164-4.045, *P*=0.015).

### Safety profiles

The safety outcomes of the two treatment groups during neoadjuvant therapy are summarized in **Table 4**. Overall, treatment-related adverse events (TRAEs) were reported in 63.9% of patients in the ICIs + TNT group and 76.4% in the ICIs + nCRT group. Grade ≥ 3 TRAEs were more frequent in the ICIs + TNT group, affecting 16.9% (15/89) of patients, compared to 9.0% (11/122) in the ICIs + nCRT group. The most common grade 3-4 TRAEs included decreased lymphocyte count (6.7% vs. 4.1%) and thrombocytopenia (4.5% vs. 2.5%) in the ICIs + TNT and ICIs + nCRT groups, respectively. Moreover, grade 3-4 diarrhea occurred in 3.4% (3/89) of patients in the ICIs + TNT group. The immune-related AEs were comparable between the two groups, with similar incidences of pruritus (8.2% vs. 9.0%), colitis (6.7% vs. 7.4%), and dermatitis (4.5% vs. 5.7%).

**Table 4:** Summary of adverse events during neoadjuvant therapy.

Adverse events	ICIs+TNT (n=89)			ICIs+nCRT (n=122)		
	Any grade	Grade 1-2	Grade 3-4	Any grade	Grade 1-2	Grade 3-4
Treatment-related adverse events	68 (76.4%)	54 (60.7%)	15 (16.9%)	78 (63.9%)	67 (54.9%)	11 (9.0%)
Nausea	14 (15.7%)	14 (15.7%)	0 (0.0%)	15 (12.3%)	15 (12.3%)	0 (0.0%)
Fatigue	18 (20.2%)	18 (20.2%)	0 (0.0%)	26 (21.3%)	26 (21.3%)	0 (0.0%)
Radiation proctitis	7 (7.9%)	7 (7.9%)	0 (0.0%)	13 (10.7%)	12 (9.8%)	1 (0.8%)
Vomiting	13 (14.6%)	12 (13.5%)	0 (1.1%)	10 (8.2%)	10 (8.2%)	0 (0.0%)
Abdominal pain	11 (12.4%)	11 (12.4%)	0 (0.0%)	14 (11.5%)	14 (11.5%)	0 (0.0%)
Anal pain	7 (7.9%)	7 (7.9%)	0 (0.0%)	14 (11.5%)	14 (11.5%)	0 (0.0%)
Diarrhea	31 (34.8%)	28 (31.5%)	3 (3.4%)	34 (27.9%)	34 (27.9%)	0 (0.0%)
Poor appetite	8 (9.0%)	8 (9.0%)	0 (0.0%)	7 (5.7%)	7 (5.7%)	0 (0.0%)
Anemia	12 (13.5%)	11 (12.4%)	1 (1.1%)	14 (11.5%)	14 (11.5%)	0 (0.0%)
Thrombocytopenia	29 (32.6%)	24 (27.0%)	4 (4.5%)	28 (23.0%)	25 (20.5%)	3 (2.5%)
Leukopenia	18 (20.2%)	17 (19.1%)	6 (6.7%)	41 (33.6%)	36 (29.5%)	5 (4.1%)
Neutropenia	30 (33.7%)	26 (29.2%)	1 (1.1%)	24 (19.7%)	23 (18.9%)	1 (0.8%)
AST or ALT elevation	8 (9.0%)	8 (9.0%)	0 (0.0%)	17 (13.9%)	16 (13.1%)	1 (0.8%)
Hand-foot syndrome	4 (4.5%)	4 (4.5%)	0 (0.0%)	3 (2.5%)	3 (2.5%)	0 (0.0%)
Immune-related adverse events	15 (16.9%)	14 (15.7%)	1 (1.1%)	18 (14.7%)	17 (13.9%)	1 (0.8%)
Pruritus	7 (7.9%)	7 (7.9%)	0 (0.0%)	11 (9.0%)	11 (9.0%)	0 (0.0%)
Dermatitis	4 (4.5%)	4 (4.5%)	0 (0.0%)	7 (5.7%)	7 (5.7%)	0 (0.0%)
Hypothyroidism	2 (2.2%)	2 (2.2%)	0 (0.0%)	1 (0.8%)	1 (0.8%)	0 (0.0%)
Colitis	6 (6.7%)	5 (5.6%)	0 (0.0%)	9 (7.4%)	13 (10.7%)	1 (0.8%)
Hyperthyroidism	0 (0.0%)	0 (0.0%)	0 (0.0%)	2 (1.6%)	2 (1.6%)	0 (0.0%)
Pneumonia	1 (1.1%)	0 (0.0%)	1 (1.1%)	0 (0.0%)	0 (0.0%)	0 (0.0%)

Postoperative complications occurred in 22.5% (20/89) of patients in the ICIs + TNT group and 18.9% (23/122) in the ICIs + nCRT group. Grade 3-4 complications were observed in eight and nine patients from the groups, respectively, with 11 of 17 affected individuals requiring a second surgery. In the ICIs + TNT group, grade 3 complications included five (5.6%) cases of adhesive intestinal obstruction, and one case each of (1.1%) rectovaginal fistula, anastomotic fistula, and abdominal infection. Conversely, the ICI+nCRT group reported four instances of adhesive intestinal obstruction (3.3%), two each of anastomotic fistula (1.6%) and abdominal hemorrhage (1.6%), and one case of anal fistula (0.8%).

### Survival outcomes

**Figure 4:**
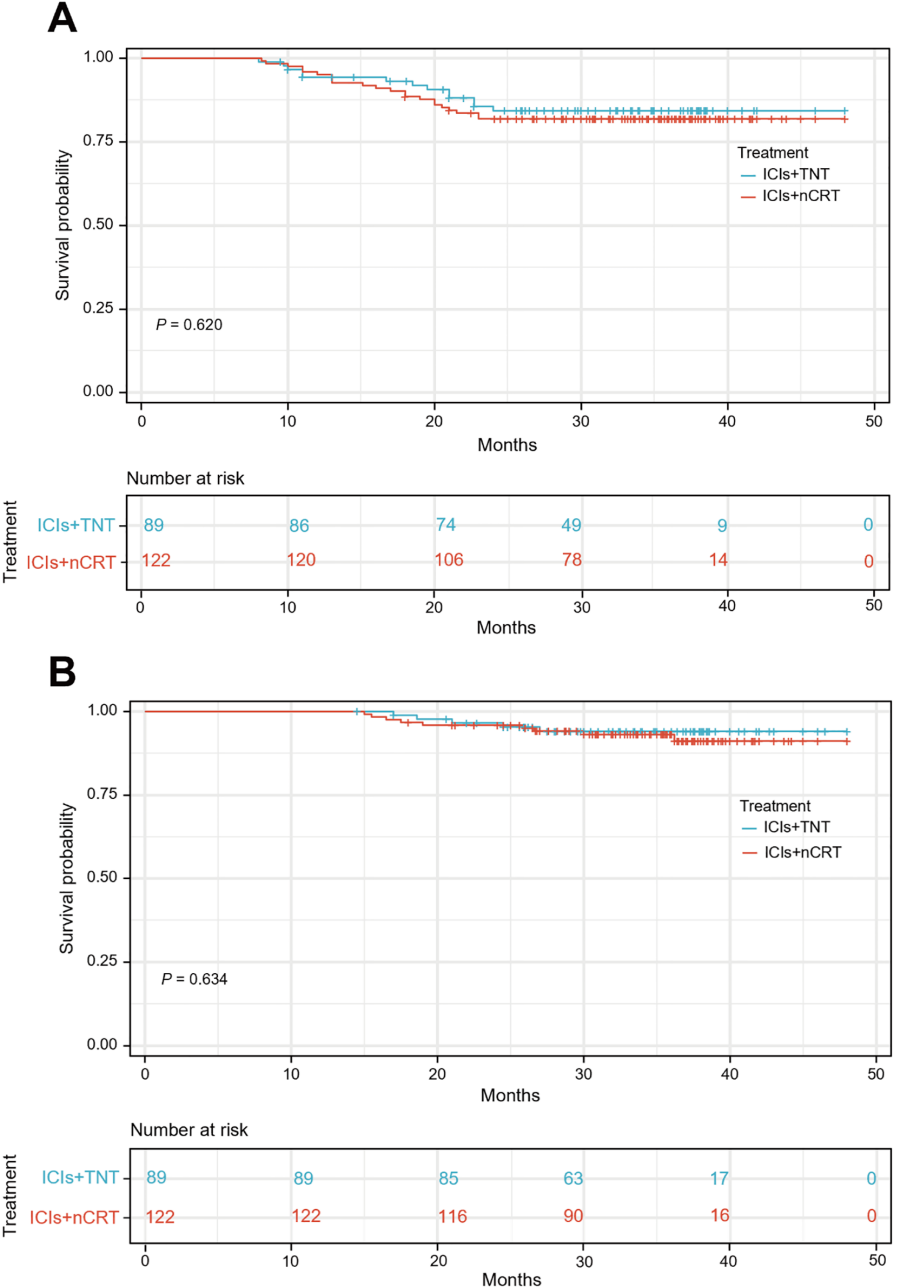
Kaplan-Meier curves for the 3-year disease-free survival **(A)** and overall survival **(B)** between ICIs + TNT group and ICIs + nCRT.

The median follow-up duration was 34.90 months (IQR, 28.05-38.40) for the ICIs + TNT group and 34.50 months (IQR, 29.50-38.05) for the ICIs + nCRT group. Long-term survival outcomes were comparable between the two groups, with similar 3-year DFS and OS rates. In the overall cohort, the 3-year DFS rate was 82.9% (95% CI: 77.8%-88.0%), and the OS rate was 92.4% (95% CI: 88.5%-96.3%). Specifically, the 3-year DFS rates were 84.3% (95% CI: 76.5%-92.1%) in the ICIs + TNT group and 81.9% (95% CI: 75.0%-88.8%) in the ICIs + nCRT group (*P*=0.620). The 3-year OS were 94.0% (95% CI: 88.9%-99.1%) and 91.1% (95% CI: 86.0%-96.2%) in the two groups, respectively (*P*=0.634). Survival curves for both groups are presented in **Figure 4**.

**Figure 5:**
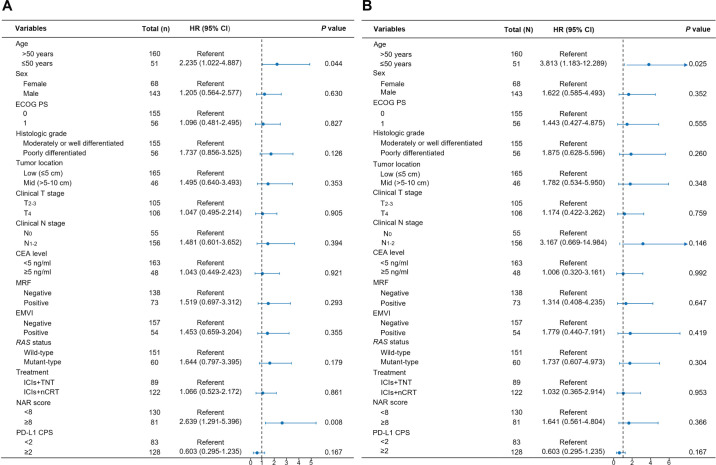
Multivariable Cox analysis of factors associated with disease-free survival **(A)** and overall survival **(B)**.

In the univariate analysis, age, mesorectal fascia (MRF) involvement, extramural venous invasion (EMVI), the neoadjuvant rectal (NAR) score, and PD-L1 CPS score were significantly associated with DFS. In contrast, age was the only factor substantially linked to OS (**Supplementary Table 2**). In the multivariate regression analysis, independent predictors of DFS included age and NAR score (**Figure 5A**). Similarly, age emerged as an independent factor influencing OS following neoadjuvant immunotherapy (**Figure 5B**). Patients aged ≤50 years exhibited a 2.235-fold higher risk of tumor recurrence compared to those aged >50 years (95% CI: 1.022-4.887). Furthermore, patients with a NAR score of <8 had considerably reduced DFS rates compared to those with a score ≥8 (HR: 2.639, 95% CI: 1.291-5.396). Regarding OS, patients aged >50 years demonstrated a 3.813-fold increased risk of mortality compared to those aged ≤50 (95% CI: 1.183-12.289).

## Discussion

In the current study, we presented the first study to report the efficacy and safety of ICIs + TNT and ICIs + nCRT strategy in pMMR/MSS LARC. The study analyzed the differences in tumor downstaging (assessed by pCR and ypT0-2), long-term survival outcomes, and sphincter preservation rates between two neoadjuvant immunotherapy regimens. The ICIs + TNT regimen demonstrated superior pCR and tumor downstaging rates (ypT0-2). However, no significant differences were observed between the two regimens regarding 3-year DFS, 3-year OS, or sphincter preservation rates.

Several studies have explored the use of chemoradiotherapy combined with immunotherapy for patients with MSS/pMMR rectal cancer. Recent prospective phase II trials have reported pCR rates ranging from 30% to 50% (12, 14, 15), with most toxicities classified as grade 1 or 2, indicating a favorable safety profile. Among these regimens, ICIs + nCRT has emerged as one of the most extensively studied neoadjuvant approaches. For instance, a prospective phase II trial by Zhang et al. (15) demonstrated that combining tislelizumab with chemoradiotherapy achieved a pCR rate of 40%, nearly three times higher than that of traditional nCRT regimens. The ICIs + nCRT significantly reduced the risk of local recurrence and potentially improved survival in patients with LARC. The AVANA trial, the largest clinical trial of neoadjuvant immunotherapy for LARC, evaluated the concurrent use of avelumab with chemoradiotherapy. This trial reported a low incidence of grade ≥3 toxicities, at just 4.0% (16). Moreover, multiple studies have compared ICIs + nCRT to standard nCRT regimens. The POLARSTAR trial used standard chemoradiotherapy as a control to assess the efficacy and safety of perioperative immunotherapy, and determine the optimal sequencing of combination therapy. Results showed that both concurrent and sequential tislelizumab with nCRT yielded higher pCR rates than chemoradiotherapy alone. Furthermore, no significant differences were observed among the groups regarding disease progression rates, grade 3-4 TRAEs, or postoperative complications (17). Similarly, other comparative studies have found ICIs + nCRT to exhibit safety and postoperative outcomes comparable to traditional chemoradiotherapy (14, 18). Several researchers have investigated alternative neoadjuvant immunotherapy approaches, including the ICIs + TNT regimen. Phase II/III clinical trials have demonstrated that ICIs + TNT achieves a favorable pCR rate and maintains an acceptable safety profile in MSS/pMMR LARC (11, 19, 20). However, most available studies are single-arm trials; direct comparisons between ICIs + TNT and ICIs + nCRT are limited. Consequently, the optimal neoadjuvant immunotherapy approach for these patients remains unclear.

While immunotherapy has proven effective in the systemic treatment of MSI-H/dMMR rectal cancer (21–23), MSS/pMMR tumors, which constitute 90%-95% of rectal cancers, are generally resistant to immune checkpoint blockade. Combining immunotherapy with other treatment modalities may enhance tumor responses in MSS/pMMR rectal cancer. For instance, radiotherapy can induce immunogenic cell death (ICD) in tumor cells, triggering pro-inflammatory signals, activating anti-tumor T cells, and recruiting tumor-infiltrating lymphocytes (TILs) (24, 25). Furthermore, radiotherapy and ICIs can modulate the tumor microenvironment, reduce immunosuppression, and stimulate the production of T cell-derived anti-tumor cytokines (26–28). These mechanisms work in tandem to produce both local and systemic synergistic effects between radiotherapy and immunotherapy. The PRECAM study demonstrated that the addition of envafolimab to short-course nCRT significantly improved pCR rate (62.5%), accompanied by acceptable mild adverse events, indicating the benefit of the addition of ICIs to nCRT for MSS/pMMR rectal cancer (29). Consistent with PRECAM study, our study also dedicated focus on MSS/pMMR tumor patients, showing that when combined with PD-1 antibody, TNT-like chemoradiotherapy resulted in better pCR rates in comparison with concurrent chemoradiotherapy with a PD-1 antibody. Specifically, the pCR rate increased substantially from 35.3% in the ICIs + nCRT group to 49.4% in the ICIs + TNT group (*P*=0.039), with a relative risk (RR) of 1.796 (95% CI 1.029-3.137). Reported pCR rates for neoadjuvant immunotherapy in prior studies have shown considerable variability, ranging from 33.3% to 50.0% (11, 12) for ICIs + TNT and 23.0% to 40.0% (15, 16) for ICIs + nCRT. In a recent meta-analysis of treatment outcomes that included pCR in rectal cancer, 13 studies with a total of 582 patients were analyzed (30). Neoadjuvant immunotherapy combined with radiotherapy was associated with higher rates of pCR and pooled major pathological response (MPR). While previous research generally indicates higher pCR rates for ICIs + TNT compared to ICIs + nCRT trials, comparisons remained constrained due to variations in clinicopathologic characteristics across trials and the absence of long-term follow-up data. In the present study, the baseline characteristics of both groups were well-matched, minimizing intergroup variability.

Achieving pCR is an established favorable prognostic marker in patients undergoing nCRT (1, 31–33). However, its predictive value for long-term prognosis in the context of neoadjuvant immunotherapy has yet to be confirmed. Our findings revealed better 3-year DFS and OS in patients who achieved pCR and had higher PD-L1 CPS (**Supplementary Figure S1**); however, no considerable differences in long-term outcomes were observed when comparing ICIs + TNT to ICIs + nCRT. Specifically, the 3-year OS rate for the ICIs + TNT group was 94.0%, aligning with survival results reported in the NRG-GI002 trial (34). Although this study did not demonstrate differences in long-term outcomes, the ICIs + TNT treatment regimen may offer more significant potential for non-operative management options in patients with LARC and MSS/pMMR. This study observed a higher proportion of patients with clinical T4 and N2 stages in the ICIs + TNT group compared to the ICIs+nCRT group. Moreover, the statistical analyses did not include potential confounders associated with poorer outcomes in the ICIs + TNT group, such as treatment compliance or lateral lymph node metastasis.

Consistent with previous findings, this study confirmed that both ICIs + TNT and ICIs + nCRT regimens were generally safe, with most AEs classified as grade 1-2 (11, 12, 16, 35). The incidence of grade ≥3 toxicities (16.9%) in the ICIs + TNT group was substantially lower than the rates reported in the PKUCH-R04 (36.0%) and TORCH trials (Group A:45.2%; Group B: 42.4%) (11, 12). Specifically, the incidences of grade 3 toxicities related to decreased platelet count were 4.5% and 2.5% in the two groups, respectively. Furthermore, all cases of reduced platelet count were resolved with timely platelet-boosting therapy, with no long-term complications or severe outcomes. An increased incidence of decreased platelet count was observed in patients undergoing platinum- and gemcitabine-based chemotherapy regimens (36, 37). However, platelet reduction associated with ICIs remains relatively rare. A meta-analysis involving 9,324 patients with cancer reported a 2.8% incidence of decreased platelet count associated with immunotherapy (38). While the precise mechanism underlying this phenomenon in neoadjuvant therapy remains unclear, it may involve the hematologic toxicity of platinum, T-cell activation, and the immune checkpoint blockade’s effects.

Age has been reported to potentially affect ICIs response rates (39); however, controversy remains regarding the association between age and antitumor efficacy of ICIs. Our findings were similar to those of an earlier study by Zhang et al. (40), but differ from a study performed by Wang et al. (41), who did not report a difference of survival outcomes in older and younger patients. The different selection criteria of the above studies may partly explain the conflicting results. A study reports that regulatory T cells are more abundant in the tumor microenvironment in younger patients, which negatively affects the efficacy of ICIs (42). In addition, a preclinical study reported that memory CD8+ T cells specifically increased with age (43). The fact that CD8^+^ T cells trigger expansion and exhibit cytolytic activity in response to immunotherapy might partly explain the better efficacy of ICI in elderly patients.

To the best of our knowledge, this study is the first to compare different neoadjuvant immunotherapy regimens in a multi-center cohort of patients with LARC. However, several limitations should be acknowledged. First, as a retrospective cohort analysis, the study is inherently prone to biases. Second, the relatively limited sample size and potential selection bias in treatment options and surgical procedures may restrict the generalizability of the findings. More extensive, prospective studies with extended follow-up are necessary to validate and explore differences in survival outcomes between the two regimens. Third, in this study, there was a 57.8% reduction in lymph node metastasis after neoadjuvant immunotherapy, including a reduction in “true” lymph node metastasis due to neoadjuvant therapy, as well as a reduction in “natural” lymph node metastasis due to high preoperative cN staging. This suggests that a large part of the decrease in lymph node metastasis after neoadjuvant therapy is attributable to inaccurate preoperative staging rather than a true treatment response. The relatively high inconsistency in cN staging results in a serious prognostic impact on the accuracy of achieving pCR. Therefore, assessing the true effect of neoadjuvant immunotherapy by improving preoperative lymph node staging is important to optimize individualized treatment regimens.

## Conclusion

In conclusion, our findings suggest that ICIs + TNT and ICIs + nCRT are comparable in terms of toxicity and perioperative complications. While ICIs + TNT demonstrated numerically higher rates of pCR and tumor downgrading (ypT0-2), it was not significantly associated with improved 3-year DFS or OS compared to ICIs + nCRT. Further prospective clinical trials, alongside refined patient selection and treatment protocols, are crucial to validate these results and establish the optimal neoadjuvant immunotherapy strategy to enhance outcomes for patients with LARC.

## Data Availability

The raw data supporting the conclusions of this article will be made available by the authors, without undue reservation.
