# Supplementary material for: Comparison of immunotherapy based total neoadjuvant therapy or standard neoadjuvant chemoradiation for locally advanced rectal cancer: a multi-institutional retrospective study
Source: Front Immunol. 2025 Apr 14;16:1513716. doi: 10.3389/fimmu.2025.1513716 (PMC12034688; doi:10.3389/fimmu.2025.1513716)
Supplement: Supplementary file 1 [file DataSheet1.docx]

Supplementary Materials

Figure S1: Kaplan-Meier curves for the 3-year disease-free survival and overall survival by pCR and PD-L1 CPS. (A) DFS in pCR/Non-pCR subgroup. (B) DFS in CPS < 2 and CPS ≥2 subgroup. (C) OS in pCR/Non-pCR. (D) OS in CPS < 2 and CPS ≥2 subgroup.

Table S1: Univariate and multivariate analyses for pathological complete response rates in subgroups.

Table S2: Univariable analysis of factors associated with disease-free survival and overall survival (n=211)


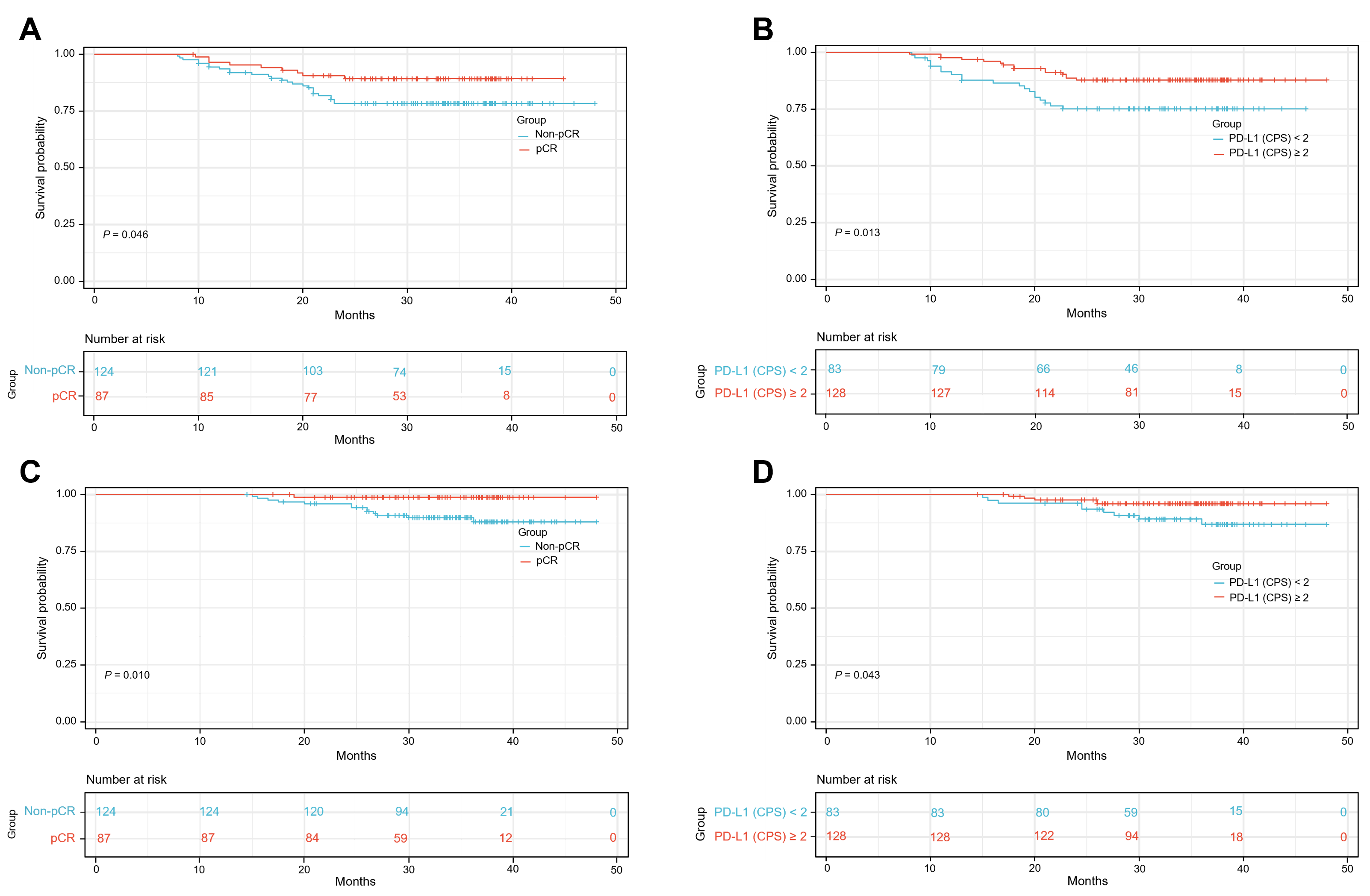


Figure S1: Kaplan-Meier curves for the 3-year disease-free survival and overall survival by pCR and PD-L1 CPS. (A) DFS in pCR/Non-pCR subgroup. (B) DFS in CPS < 2 and CPS ≥2 subgroup. (C) OS in pCR/Non-pCR. (D) OS in CPS < 2 and CPS ≥2 subgroup.

Supplementary Table 1. Univariate and multivariate analyses for pathological complete response rates in subgroups.

| **Subgroup** | **pCR, n/N (%)** | **Univariate analysis** | | **Multivariate analysis** | |
| --- | --- | --- | --- | --- | --- |
|  |  | **OR (95% CI)** | ***P* value** | **OR (95% CI)** | ***P* value** |
| Treatment group |  |  | 0.039 |  | 0.015 |
| ICIs+nCRT group | 43/122 (35.3) | Referent |  | Referent |  |
| ICIs+TNT group | 44/89 (49.4) | 1.796 (1.029-3.137) |  | 2.170 (1.164-4.045) |  |
| Age |  |  | 0.004 |  | 0.006 |
| >50 years | 75/160 (46.9) | Referent |  | Referent |  |
| ≤50 years | 12/51 (23.5) | 0.369 (0.170-0.715) |  | 0.320 (0.142-0.725) |  |
| Sex |  |  | 0.773 |  | 0.500 |
| Female | 29/68 (42.6) | Referent |  | Referent |  |
| Male | 58/143 (40.6) | 0.918 (0.511-1.647) |  | 0.799 (0.415-1.536) |  |
| ECOG PS |  |  | 0.197 |  | 0.145 |
| 0 | 68/155 (43.9) | Referent |  | Referent |  |
| 1 | 19/56 (33.9) | 0.657 (0.347-1.243) |  | 0.587 (0.287-1.202) |  |
| Histologic grade |  |  | 0.508 |  | 0.299 |
| Poorly differentiated | 21/56 (37.5) | Referent |  | Referent |  |
| Moderately or well differentiated | 66/155 (42.6) | 1.236 (0.660-2.315) |  | 1.463 (0.713-3.003) |  |
| Distance from the anal verge |  |  | 0.506 |  | 0.350 |
| Low (≤5 cm) | 70/165 (42.4) | Referent |  | Referent |  |
| Mid (>5-10 cm) | 17/46 (37.0) | 0.796 (0.406-1.560) |  | 0.693 (0.322-1.493) |  |
| Clinical T stage |  |  | 0.014 |  | 0.109 |
| T2 | 8/12 (66.7) | Referent |  | Referent |  |
| T3 | 45/93 (48.4) | 0.469 (0.132-1.665) | 0.241 | 0.429 (0.112-1.640) | 0.216 |
| T4 | 34/106 (32.1) | 0.236 (0.066-0.839) | 0.026 | 0.271 (0.070-1.046) | 0.058 |
| Clinical N stage |  |  | 0.460 |  | 0.276 |
| N0 | 25/55 (45.5) | Referent |  | Referent |  |
| N1-2 | 62/156 (39.7) | 0.791 (0.426-1.471) |  | 0.670 (0.326-1.377) |  |
| CEA level |  |  | 0.011 |  | 0.101 |
| <5 ng/ml | 75/163 (46.0) | Referent |  | Referent |  |
| ≥5 ng/ml | 12/48 (25.0) | 0.391 (0.190-0.805) |  | 0.505 (0.223-1.141) |  |
| MRF |  |  | 0.229 |  | 0.958 |
| Negative | 61/138 (44.2) | Referent |  | Referent |  |
| Positive | 26/73 (35.6) | 0.698 (0.389-1.254) |  | 0.981 (0.485-1.985) |  |
| EMVI |  |  | 0.296 |  | 0.768 |
| Negative | 68/157 (43.3) | Referent |  | Referent |  |
| Positive | 19/54 (35.2) | 0.711 (0.374-1.350) |  | 0.891 (0.413-1.920) |  |
| *RAS* status |  |  | 0.696 |  | 0.323 |
| Wild-type | 61/151 (40.4) | Referent |  | Referent |  |
| Mutant-type | 26/60 (43.3) | 1.128 (0.616-2.067) |  | 1.415 (0.711-2.814) |  |
| PD-L1 CPS |  |  | 0.158 |  | 0.908 |
| <1 | 19/57 (33.3) | Referent |  | Referent |  |
| ≥1 | 68/154 (44.2) | 1.581 (0.837-2.987) |  | 1.068 (0.354-3.220) |  |
| PD-L1 CPS |  |  | 0.040 |  | 0.216 |
| <2 | 27/83 (32.5) | Referent |  | Referent |  |
| ≥2 | 60/128 (46.9) | 1.830 (1.029-3.254) |  | 1.888 (0.690-5.165) |  |

Supplementary Table 2. Univariable analysis of factors associated with disease-free survival and overall survival (n=211)

| **Variables** | **Total (N)** | **Disease-free Survival** | | **Overall Survival** | |
| --- | --- | --- | --- | --- | --- |
|  |  | **HR (95% CI)** | ***P* value** | **HR (95% CI)** | ***P* value** |
| Age |  |  | 0.009 |  | 0.030 |
| >50 years | 160 | Referent |  | Referent |  |
| ≤50 years | 51 | 2.433 (1.254-4.721) |  | 2.865 (1.105-7.427) |  |
|  |  |  |  |  |  |
| Sex |  |  | 0.761 |  | 0.479 |
| Female | 68 | Referent |  | Referent |  |
| Male | 143 | 1.116 (0.549-2.269) |  | 1.418 (0.540-3.724) |  |
| ECOG PS |  |  | 0.929 |  | 0.802 |
| 0 | 155 | Referent |  | Referent |  |
| 1 | 56 | 1.035 (0.487-2.201) |  | 1.143 (0.403-3.245) |  |
| Histologic grade |  |  | 0.056 |  | 0.437 |
| Moderately or well differentiated | 155 | Referent |  | Referent |  |
| Poorly differentiated | 56 | 1.921 (0.983-3.754) |  | 1.484 (0.549-4.015) |  |
| Tumor location |  |  | 0.540 |  | 0.754 |
| Low (≤5 cm) | 165 | Referent |  | Referent |  |
| Mid (>5-10 cm) | 46 | 1.266 (0.595-2.693) |  | 1.196 (0.390-3.674) |  |
| Clinical T stage |  |  | 0.553 |  | 0.715 |
| T2-3 | 105 | Referent |  | Referent |  |
| T4 | 106 | 1.220 (0.632-2.354) |  | 1.194 (0.461-3.095) |  |
| Clinical N stage |  |  | 0.366 |  | 0.182 |
| N0 | 55 | Referent |  | Referent |  |
| N1-2 | 156 | 1.464 (0.641-3.342) |  | 2.734 (0.625-11.958) |  |
| CEA level |  |  | 0.464 |  | 0.569 |
| <5 ng/ml | 163 | Referent |  | Referent |  |
| ≥5 ng/ml | 48 | 1.313 (0.633-2.723) |  | 1.355 (0.477-3.846) |  |
| MRF |  |  | 0.046 |  | 0.624 |
| Negative | 138 | Referent |  | Referent |  |
| Positive | 73 | 1.943 (1.011-3.734) |  | 1.298 (0.457-3.686) |  |
| EMVI |  |  | 0.045 |  | 0.425 |
| Negative | 157 | Referent |  | Referent |  |
| Positive | 54 | 1.987 (1.016-3.884) |  | 1.661 (0.477-5.782) |  |
| *RAS* status |  |  | 0.067 |  | 0.223 |
| Mutant-type | 151 | Referent |  | Referent |  |
| Wild-type | 60 | 1.857 (0.957-3.603) |  | 1.823 (0.694-4.790) |  |
| Treatment |  |  | 0.780 |  | 0.963 |
| ICIs+TNT | 89 | Referent |  | Referent |  |
| ICIs+nCRT | 122 | 1.100 (0.563-2.150) |  | 1.023 (0.389-2.688) |  |
| NAR score |  |  | 0.003 |  | 0.205 |
| <8 | 130 | Referent |  | Referent |  |
| ≥8 | 81 | 2.758 (1.411-5.392) |  | 1.851 (0.714-4.797) |  |
| PD-L1 CPS |  |  | 0.016 |  | 0.054 |
| <2 | 83 | Referent |  | Referent |  |
| ≥2 | 128 | 0.438 (0.224-0.855) |  | 0.341 (0.114-1.016) |  |
